# The role of calprotectin in giant cell arteritis - from pathophysiology to possible clinical applications

**DOI:** 10.3389/fimmu.2025.1608402

**Published:** 2025-08-13

**Authors:** Emilia Kudraszew, Leszek Roszkowski, Marzena Ciechomska, Jakub Wroński

**Affiliations:** ^1^ Department of Rheumatology, National Institute of Geriatrics, Rheumatology and Rehabilitation, Warsaw, Poland; ^2^ Department of Outpatient Clinics, National Institute of Geriatrics, Rheumatology and Rehabilitation, Warsaw, Poland; ^3^ Department of Pathophysiology and Immunology, National Institute of Geriatrics, Rheumatology and Rehabilitation, Warsaw, Poland

**Keywords:** giant cell arteritis, calprotectin, biomarker, disease activity, diagnosis

## Abstract

Giant cell arteritis (GCA) is an immune-mediated vasculitis predominantly affecting individuals aged 50 years and older, with clinical manifestations often overlapping with polymyalgia rheumatica (PMR). Despite advances in imaging and the advent of novel steroid-sparing agents, such as tocilizumab, challenges persist in accurately diagnosing and monitoring disease activity. Traditional inflammatory markers like C-reactive protein and erythrocyte sedimentation rate are frequently limited by their inability to fully capture disease dynamics, especially in patients receiving IL-6 inhibitors. In this context, calprotectin (CLP), a heterodimeric complex derived from S100A8/S100A9 proteins, has emerged as a promising biomarker due to its integral role in mediating inflammatory responses and its relative independence from IL-6 pathways. This review synthesizes current evidence on the biological functions of CLP in GCA pathogenesis, its potential utility in distinguishing between different clinical forms of the GCA-PMR spectrum, and its role in assessing disease activity and guiding therapeutic decisions. Furthermore, emerging CLP-targeted therapies in other inflammatory conditions may offer novel treatment avenues for GCA. Future research should focus on validating CLP as a predictive marker for relapse and refining its integration into clinical monitoring protocols to enhance patient outcomes.

## 1 Introduction

Giant cell arteritis (GCA) is an immune-mediated ischemic condition and is the most common form of systemic vasculitis in patients aged ≥50 years (1). It belongs to the group of large vessel vasculitis according to the 2012 International Chapel Hill Consensus Conference nomenclature (2). Previously known as temporal arteritis, which is now considered a form of GCA, the disease itself is now viewed more broadly. So far, no uniform classification of the disease has been established. Currently, more and more authors are inclined to the view that we should speak of a GCA-polymyalgia rheumatica (PMR) disease spectrum (3), distinguishing between cranial GCA, extracranial GCA, generalized inflammation, and PMR. It should be noted that nearly 50% of GCA patients exhibit PMR clinical presentations, while nearly 20% of PMR patients also have concomitant GCA manifestations (4). Glucocorticoids (GCs) have long been the treatment of choice for GCA, but recent studies have shown that additional agents such as tocilizumab, methotrexate, and leflunomide are effective steroid-sparing alternatives (5–7).

Recent advancements in imaging technologies, classification criteria, and novel therapies have improved diagnosis and treatment. However, there are still many unknowns regarding relapse monitoring and long-term treatment strategies. In patients with GCA in clinical remission, long-term clinical monitoring is strongly recommended by the 2021 ACR guidelines for the management of giant cell arteritis (5). This is the only strong recommendation in this paper, reflecting both the minimal risk of routine surveillance and the potentially severe consequences of inadequate monitoring – namely aneurysmal dissection or rupture, and vascular stenosis leading to ischemia. The optimal frequency and duration of monitoring remain unknown. Clinical monitoring may include history, physical examination, and laboratory and imaging studies. Imaging is becoming increasingly important in diagnosing and assessing disease activity. Arterial ultrasound, computed tomography (CT), magnetic resonance imaging (MRI), and 18F-fluorodeoxyglucose positron emission tomography/computed tomography (18F-FDG PET/CT) are used for this purpose; however, all of them have limitations, and when used alone, they are not always sufficient for this purpose (8).

Laboratory monitoring of GCA is based on the evaluation of traditional inflammatory markers such as C-reactive protein (CRP) and erythrocyte sedimentation rate (ESR). According to ACR guidelines, if inflammatory markers are elevated alone, clinical observation and monitoring without intensification of immunosuppressive treatment are conditionally recommended. This may justify more frequent clinical check-ups and the use of imaging studies (4). The BSR guidelines differ in this respect, making the reduction of glucocorticoid (GC) doses dependent on the normalization of inflammatory markers (6). EULAR guidelines are rather vague on this matter. Cases of elevated inflammatory markers alone, suggest observation and monitoring rather than immediate intensification of immunosuppressive therapy unless additional clinical or imaging signs suggesting disease activity occur (7).

An additional challenge is that the use of IL-6 inhibitors alters the natural course of inflammation, making it more difficult to track the true state of the disease using traditional inflammatory markers and clinical images. IL-6 inhibitors can significantly reduce symptoms of inflammation, such as fever, pain, and changes in laboratory test results (e.g., CRP).

As a result, there is an ongoing search for biomarkers of GCA activity. New blood biomarkers that are less dependent on the IL-6 axis, such as IL-23, B cell-activating factor, osteopontin, and calprotectin, are being investigated. One of the most promising markers is serum calprotectin (CLP) (9). CLP is a heterodimeric complex (S100A8/S100A9, also known as myeloid-related protein 8/14 [MRP8/14]) formed by two binding proteins belonging to the S100 protein family and is produced mostly by neutrophils, monocytes, and early differentiated macrophages (10). CLP is a recognized marker in inflammatory bowel diseases (IBD). Determination of fecal calprotectin concentration is helpful both in diagnosing and monitoring IBD activity. However, its important biological role in inflammation, immune cell recruitment, and tissue damage, which are processes that underlie vascular injury, means that it may also find application in GCA and other vasculopathies (10, 11). In this article, we discussed the biological role of CLP in GCA, taking into account the pathophysiology of the disease, as well as literature data and perspectives on the clinical utility of calprotectin in diagnostics, differentiation of clinical forms, and monitoring disease activity of GCA.

## 2 Biological role of calprotectin in GCA and other vasculitis

**Figure 1:**
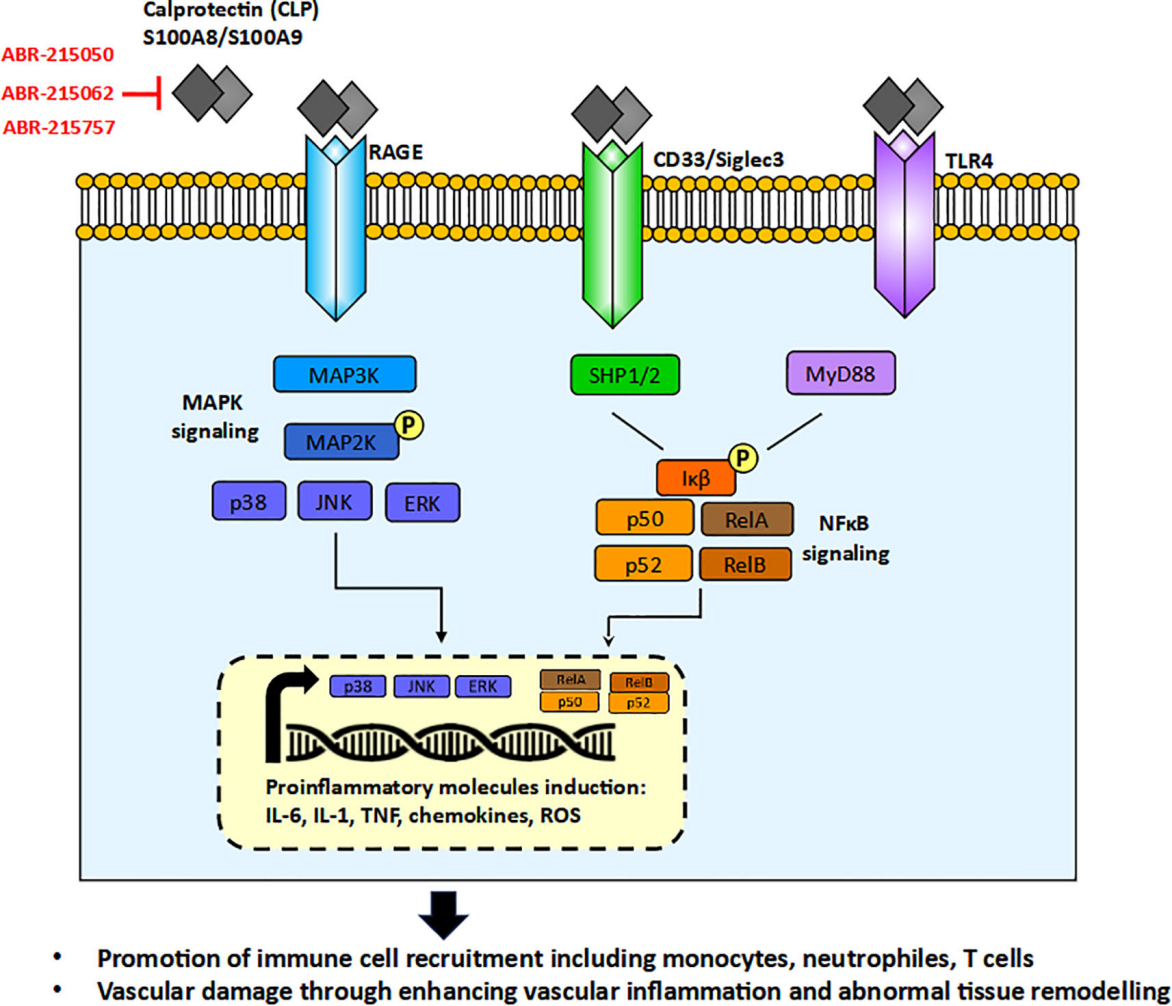
The role of calprotectin (CLP) in the pathogenesis of Giant Cell Arteritis (GCA) through MAPK (Mitogen-Activated Protein Kinase) and NF-κB (Nuclear Factor kappa-light-chain-enhancer of activated B cells) signaling pathways, and the potential mechanisms of CLP-neutralizing therapies. ABR-215050, ABR-215062, ABR-215757 orally available molecules blocking CLP activity.

The S100 family, to which CLP belongs, comprises 25 proteins in humans containing a specific Ca²^+^-binding motif (helix-loop-helix), called the EF-hand, which tightly responds to Ca²^+^ levels and is thus exclusively implicated in intracellular and extracellular regulatory activities (10). Extracellularly released CLP can be involved in inflammation, immune cell recruitment, and tissue damage, all contributing to the vascular damage seen in the pathogenesis of GCA and other vascular diseases (10). Many studies demonstrated elevated levels of CLP in sera of GCA patients (12–15). A summary of the possible role of CLP in the pathogenesis of GCA is presented in **Figure 1**.

Secreted CLP functions as a Damage-associated Molecular Pattern (DAMP) or alarmin, released in response to cellular stress or tissue injury, activating the innate immune system during non-infectious inflammatory processes. CLP interacts with a variety of receptors, including Toll-like receptor 4 (TLR4), the receptor for advanced glycation end products (RAGE), and CD33. This interaction leads to the activation of key signaling pathways, such as MAPK/NF-κB and AP-1, culminating in the production of proinflammatory cytokines, including IL-6, TNF, and IL-1, chemokines and generation of reactive oxygen species (ROS) (11). These molecules are notably elevated in GCA and other forms of vasculitis. The receptors for CLP are expressed on various innate immune cells, such as neutrophils, monocytes/macrophages, and dendritic cells (DCs), as well as on non-immune cells, including endothelial cells and smooth muscle cells that are critical for blood vessel formation. To date, no studies have investigated the expression of RAGE or CD33 in patients with GCA, and the available study on TLR4 did not demonstrate a difference in its expression between GCA patients and controls (16). Furthermore, TLR4 gene polymorphisms were not associated with the occurrence of GCA itself nor did they predispose to any of the clinical forms of GCA (17, 18). This may suggest that only disturbed levels of CLP but not their receptors play a role in GCA pathogenesis.

Recent evidence has highlighted a previously underappreciated role for neutrophils in the pathogenesis of GCA. Immunohistochemistry staining directly demonstrated co-expression between CLP and markers for macrophages (CD68 and CD163) and neutrophils (CD15) in temporal artery biopsy (TAB) of GCA patients, suggesting an important role of innate immune cells in initiating inflammatory responses which results in inappropriate tissue remodeling (12, 13). CLP is also present in neutrophil extracellular traps (NETs) - DNA-protein structures released by neutrophils that trap pathogens, but can also promote inflammation and tissue damage. Studies have demonstrated NETs in GCA patients near the vasa vasorum (19). NETs may promote immune-mediated thrombosis and tissue injury in GCA, as intraluminal thrombosis is present in up to 20% of positive biopsies (20). Moreover, increased circulating NETs in GCA patients were also found, supporting systemic neutrophil activation (21). However, the functional relevance of CLP in NETs remains to be fully elucidated, warranting further investigation.

The role of T cells in the pathogenesis of GCA is well established (20, 22, 23). CLP may represent a mechanistic link between innate and adaptive immunity through its effects on T cells. Upon binding to its receptors on innate immune cells CLP promotes the recruitment and activation of T cells within the arterial wall. The previously described effect of CLP on the production of proinflammatory cytokines, including IL-6, TNF-α, and IL-1β, primarily via activation of the NF-κB pathway, influences T cells. These cytokines play a role in T cell recruitment and differentiation, particularly the polarization of naïve CD4^+^ T cells toward Th1 and Th17 phenotypes, seen in the vascular lesions of GCA (20, 22). The aforementioned interaction of CLP with TLR4 also contributes to T-cell response, as evidenced in murine models showing increased expression of markers related to T-cell recruitment (TCR) and activation (CD40L, LTα, IFN-γ) (24). Additionally, CLP contributes to the induction of autoreactive CD8^+^ T cells by acting as a costimulatory enhancer alongside CD40/CD40L signaling, thereby promoting loss of T cell tolerance (25). The regulatory effects of CLP, however, are cell-type dependent: DCs from S100A9-deficient mice exhibit an exaggerated proinflammatory profile and an enhanced capacity to induce T cell proliferation, while macrophages lacking S100A9 show no such differences in their response to TLR ligands (26). Moreover, CLP acts as an endogenous ligand for CD69 on regulatory T cells, supporting their differentiation and contributing to the maintenance of immune homeostasis (27).

## 3 Clinical utility of calprotectin in autoimmune inflammatory rheumatic diseases

Due to its important role in the pathogenesis of autoimmune inflammatory rheumatic diseases (AIIRD) and the fact that CLP is easily detectable in blood serum and synovial fluid, it has recently gained prominence as a promising biomarker for diagnosis, assessment of disease activity, and predicting treatment response/disease relapse in an increasing number of AIIRDs (28, 29).

### 3.1 Studies of calprotectin in inflammatory arthritis

Among all rheumatic diseases, the association of CLP and rheumatoid arthritis (RA) is the most extensively studied and best understood. Studies confirm the correlation of CLP concentration with disease activity, CRP levels, and synovitis detected by ultrasound (30). Our studies also confirmed that CLP is a good marker of RA activity that can be regulated by epigenetic drugs related to DNA methylation such as GCs or RG108 (31). Furthermore, CLP levels were independently associated with radiological progression in RA, with high CLP levels in the early stages of the disease predicting future erosive damage, as well as with the response to drug therapies (32, 33). In patients with SpA, several studies have shown higher levels of CLP compared to the healthy control group (HC), particularly in polyarticular and peripheral disease with large joint involvement (34). Additionally, the correlation of CLP levels with disease activity scores and ultrasound findings has been confirmed in patients with SpA (35).

### 3.2 Studies of calprotectin in systemic vasculitis other than GCA

Numerous studies have also been conducted on patients with various types of vasculitis. It has been observed that elevated levels of CLP in the serum of patients with Kawasaki disease (KD) may predict the severity of vascular inflammation (36). Increased levels of CLP may also persist in plasma for months to decades after the acute phase of KD, suggesting a lasting, subclinical inflammatory state (37). Also in children with IgA vasculitis serum CLP levels correlated with disease activity, C-reactive protein, complement component C3, ferritin, and fibrinogen. Additionally, CLP levels were higher in patients with larger areas of skin covered by rash (38).

In patients with anti-neutrophil cytoplasmic antibodies (ANCA)–associated vasculitis (AAV) an increase in serum CLP levels during the clinical remission phase indicates a subgroup with a higher risk of relapse, identifying those patients who require more intensive or prolonged treatment (39, 40). It was also shown that in AAV the deterioration of kidney function, hematuria, increasing proteinuria, and unchanged levels of ANCA correlated with higher levels of CLP in serum (41). In another study, an increase in the CLP level was confirmed to be an independent predictor of renal function decline (40). This proves that CLP during remission in AAV can be useful for identifying subclinical inflammatory states and patients with poorer renal prognosis, and may therefore be useful for intensifying treatment in such patients. However, different study of AAV and large vessel vasculitis (LVV) showed that CLP levels were elevated in patients with vasculitis compared to HC, but not associated with disease activity. Still, due to the high heterogeneity of the study group of patients – it involved granulomatosis with polyangiitis, microscopic polyangiitis, Takayasu disease (TAK), and GCA – and the use of different therapeutic regimes that may have a negative impact on the suppressed inflammatory response, this study has limitations (15). However, it should be remembered that despite the common denominator in the form of vasculitis, the pathophysiology of AAV and LVV is different. In AAV, pathogenic ANCA activate neutrophils, leading to pauci‐immune fibrinoid necrosis of small vessels (42). By contrast, LVV is mostly characterized by T-cell and macrophage infiltration of the large vessel media, the formation of granulomas with multinucleated giant cells, and resultant intimal hyperplasia (22). Just as in AAV the CLP concentration may be directly related to the infiltration of neutrophils, in LLV CLP may rather play an indirect role in T-cell recruitment. Therefore, the results concerning CLP from AAV cannot be translated directly to LVV.

The results for one form of LVV, TAK, are more equivocal. In one study investigating the utility of serial measurement of CLP as a biomarker of clinical disease activity and angiographic progression in TAK, CLP levels were not only higher in patients with TKA than in HC but also patients with active disease had higher levels than those with stable disease. Furthermore, CLP concentrations correlated with changes in disease activity independently of GC dosage but remained unchanged in patients who were unresponsive to treatment or experienced disease relapse. What’s more, CLP levels increased during follow-up in more angiographic progressors than non-progressors (43). On the other hand, another study found no significant differences in CLP levels between active and inactive diseases (14).

## 4 Emerging clinical utility of calprotectin in GCA

As in the case of other systemic vasculitis, in recent years there has been an increasing number of studies indicating the possible clinical use of CLP in GCA - in diagnosis, including differentiation of the disease form, and as a marker of disease activity, helping to monitor the effectiveness of treatment and make therapeutic decisions. The summary of the research is presented in **Table 1**.

**Table 1:** Studies evaluating the clinical use of calprotectin in GCA.

Author and year	Study population	Result
Disease diagnosis		
Foell et al., 2004 (12)	42 GCA, 35 HC	↑ CLP in GCA vs HC
Van Sleen et al., 2019 (13)	41 GCA, 33 HC	↑ CLP in GCA vs HC
Springer et al., 2018 (14)	59 GCA, 35 HC	↑ CLP in GCA vs HC
Michailidou et al., 2022 (15)	68 GCA, 30 HC	↑ CLP in GCA vs HC
Disease stratification		
Van Sleen et al., 2019 (13)	41 GCA: 11 cranial, 8 extracranial, and 22 combined	No difference in CLP between different GCA forms
Michailidou et al., 2022 (15)	32 GCA and 36 GCA with PMR	↑ CLP in GCA alone vs GCA with PMR
Brun et al., 2005 (44)	10 GCA, 4 GCA with PMR, and 33 PMR	↑ correlation of CLP with the acute phase proteins in PMR vs GCA
Disease activity marker		
Springer et al., 2018 (14)	59 GCA	↑ serum CLP in active GCA vs inactive
Saut et al., 2023 (45)	22 GCA	↑ serum CLP in active GCA vs inactive CLP does not predict relapse
Brun et al., 2005 (44)	10 GCA, 4 GCA with PMR, and 33 PMR	CLP correlated with required GCs dose
Benucci et al., 2024 (46)	68 GCA	good correlation between CLP and the PET and US Halo scores
Michailidou et al., 2022 (15)	18 GCA with active disease paired with 18 with inactive disease	No difference in CLP between active and inactive disease

CLP, calprotectin; GCs, glucocorticoids; GCA, giant cell arteritis; HC, healthy controls; PET, positron emission tomography; PMR, polymyalgia rheumatica; US, ultrasound; ↑, higher.

### 4.1 Application in GCA diagnostics

Differentiating between different GCA-PMR spectrum forms of disease can be difficult. Such a distinction may not only be a prognostic factor for the further course of the disease, which may be associated with the need to use additional immunomodulatory drugs beyond GCs (PMR GCA overlap vs PMR alone). The distinction also indicates diagnostic methods that should be used for monitoring disease activity and vascular complications (stenosis, aneurism). CLP may be helpful in this regard. One study showed higher CLP concentrations in patients with GCA alone compared to the overlap of GCA and PMR (15). The observed differences may result from different levels of neutrophil recruitment and activation in different forms of the GCA-PMR disease spectrum but require confirmation. This may be clinically useful because patients with overlapping GCA and PMR are more likely to have extracranial GCA (47, 48), which is at risk for diagnostic and therapeutic delay (49, 50). Only one study assessed differences in CLP levels between different forms of GCA (cranial vs. extracranial vs. combined) but found no differences. However, it should be noted that the groups were small (respectively 11, 8, and 22 patients) (13). Additionally, one of the studies showed that CLP correlates more strongly with the acute phase proteins in PMR than in GCA (44). This may indicate that in GCA CLP may play a greater role as an independent marker of disease activity, which is a more widely studied issue.

### 4.2 Calprotectin as a GCA disease activity marker

In a study by Springer et al. on 59 patients with GCA, significant differences were found between serum CLP levels in patients with active disease (defined as Birmingham Vasculitis Activity Score version 1 score greater than 0 and a physician’s global assessment greater than 0) compared to inactive disease. Moreover, a prediction model of active disease based on CLP, CRP, and ESR was found to be better than a model based on CRP and ESR alone (14). Similar results were observed in a group of 22 patients followed for one year. CLP levels were significantly higher in active disease (defined as present GCA-related symptoms and elevated acute phase reactants) compared with patients with inactive disease. If disease activity was defined as GCA-related symptoms only, regardless of acute phase reactants, the difference was still significant. However, CLP levels did not predict relapse (45). What’s more, in a retrospective study of 47 patients with GCA-PMR spectrum disease, the authors showed that in patients who had received higher doses of GCs, and therefore most likely had a more active disease requiring more intensive treatment, higher CLP concentrations were also noted (44). One of the most interesting studies recently presented an observational study of 68 patients with GCA in which CLP levels were checked in patients where disease activity was monitored by imaging studies in ultrasound and 18F-FDG PET/CT. The study showed a good correlation between CLP and the PET and Halo scores (46).

Different results were presented in a study involving 18 patients with GCA, where the CLP level did not correlate with disease activity assessed by the physician global assessment scale (15). This study also found no differences in CLP levels between patients in remission and those with active disease, although the methodology did not define remission. The study authors themselves indicate that this could indicate subclinical vasculitis. A similar observation (of being a marker of subclinical vasculitis) was made by the authors of a study of 41 patients with GCA, in which CLP concentration did not decrease after GCA treatment (13). In this study, remission was defined based on clinical signs and symptoms of GCA. Therefore, the observed differences between studies may be due to differences in how disease activity was assessed – such as through physician global assessment, the Birmingham Vasculitis Activity Score, GCA-related symptoms, acute phase reactants, or imaging techniques. Some of them may indicate subclinical vasculitis. The significance of possible ongoing subclinical vasculitis remains an open question - is it an indication for intensification of therapy? So far, the guidelines do not provide clear answers to this question. Another contributing factor could be the smaller sample size in the study by Michailidou et al., compared to studies that demonstrated a correlation between CLP levels and disease activity. This limited sample size may have hindered the detection of statistically significant differences. Additionally, differences in CLP measurement methodology must be considered. In some studies, CLP was measured in serum (14, 45), while in others it was assessed in plasma (15). In the study by Benucci et al., the biological material used for CLP determination was not specified (46). Measurement of CLP levels in plasma may be more appropriate, as Michailidou et al. noted that coagulation processes can induce the release of calprotectin. Therefore, serum CLP levels may not reflect physiological conditions, but rather artificially elevated levels resulting from coagulation (15). This distinction is supported by findings in ulcerative colitis, a disease in which CLP is an established marker of disease activity. In that context, plasma-based measurements are considered more accurate than those performed in serum (51, 52).

Importantly, although CLP induces IL-6 production (via activation of TLR4 and RAGE and stimulation of the NF-κB signaling pathway) (11), CLP concentration does not correlate with IL-6. This makes CLP an attractive marker of disease activity among patients treated with IL-6 receptor inhibitors (13). Due to IL-6 receptor blocking, IL-6 levels are elevated in these patients. The clinical usefulness of CLP in monitoring patients treated with tocilizumab has already been proven in patients with RA, where CLP was found to be better suited to detect RA activity than ESR and CRP in patients treated with tocilizumab (53).

## 5 Therapeutic perspectives

The important biological role of CLP in the pathophysiology of GCA makes it a potential therapeutic target. Although CLP itself has not yet been a primary target in therapeutic strategies for GCA, small molecules that inhibit CLP formation have progressed to Phase 2 and 3 clinical trials in other indications. These investigational therapies target a variety of cancers and inflammatory conditions, including multiple myeloma, multiple sclerosis, prostate cancer, uveitis, and sepsis-induced myocardial dysfunction. Notable agents in this class include laquinimod (ABR-215062), tasquinimod (ABR-215050), and paquinimod (ABR-215757). For example, laquinimod (ABR-215062) has been shown to reduce proinflammatory cytokines activity and ROS formation resulting in improvement of myocardial dysfunction (54), protecting caudate volume loss in Huntington’s disease (55), and improving the effect of clinical remission in Crohn’s disease (56). Tasquinimod (ABR-215050) by blocking S100A9 activity was able to sensitize cancer cells for apoptosis (57, 58). Paquinimod (ABR-215757) was proven to inhibit skin fibrosis in systemic sclerosis patients (59) and reduce disease activity in experimental lupus (60). Overall, these examples of CLP-neutralizing therapies in cancer and inflammatory conditions may also hold promise for the future treatment of GCA, potentially leading to reduced immune cell infiltration, downregulation of proinflammatory cytokines, and inhibition of blood vessel remodeling.

## 6 Summary

GCA remains a complex immune-mediated vasculitis with evolving diagnostic and therapeutic strategies. Despite advancements in imaging, classification, and treatment, challenges persist in diagnosing, disease monitoring, and relapse prevention. CLP has emerged as a promising biomarker for GCA, with potential utility in diagnosing disease, assessing activity, and guiding treatment decisions – especially in patients receiving IL-6 inhibitors. Possible further studies evaluating the usefulness of CLP may focus on better diagnosis, e.g. evaluating the correlation of CLP with south-end giant cell arteritis probability score or CLP differential value in different forms of GCA-PMR spectrum diseases. Further research is also required on the more comprehensive assessment of disease activity with CLP, e.g. taking into account newer scales such as Large-Vessel Vasculitis Index of Damage. It would also be worthwhile to study the predictive value of CLP in the assessment of subclinical inflammation of large vessels (taking into account the latest imaging studies) and their significance, whether it predicts disease recurrence, in a larger number of patients. Expanding our understanding of CLP in GCA could enhance precision medicine approaches, ultimately improving patient outcomes.
